# Deciphering the functional role of clinical mutations in ABCB1, ABCC1, and ABCG2 ABC transporters in endometrial cancer

**DOI:** 10.3389/fphar.2024.1380371

**Published:** 2024-05-02

**Authors:** Aayushi Gupta, Manu Smriti Singh, Bipin Singh

**Affiliations:** ^1^ Centre for Life Sciences, Mahindra University, Hyderabad, India; ^2^ Interdisciplinary Centre for Nanosensors and Nanomedicine, Mahindra University, Hyderabad, India

**Keywords:** cancer, ATP-binding cassette transporters, chemoresistance, mutation, mitoxantrone, drug resistance, efflux transporters

## Abstract

ATP-binding cassette transporters represent a superfamily of dynamic membrane-based proteins with diverse yet common functions such as use of ATP hydrolysis to efflux substrates across cellular membranes. Three major transporters—P-glycoprotein (P-gp or ABCB1), multidrug resistance protein 1 (MRP1 or ABCC1), and breast cancer resistance protein (BCRP or ABCG2) are notoriously involved in therapy resistance in cancer patients. Despite exhaustive individual characterizations of each of these transporters, there is a lack of understanding in terms of the functional role of mutations in substrate binding and efflux, leading to drug resistance. We analyzed clinical variations reported in endometrial cancers for these transporters. For ABCB1, the majority of key mutations were present in the membrane-facing region, followed by the drug transport channel and ATP-binding regions. Similarly, for ABCG2, the majority of key mutations were located in the membrane-facing region, followed by the ATP-binding region and drug transport channel, thus highlighting the importance of membrane-mediated drug recruitment and efflux in ABCB1 and ABCG2. On the other hand, for ABCC1, the majority of key mutations were present in the inactive nucleotide-binding domain, followed by the drug transport channel and membrane-facing regions, highlighting the importance of the inactive nucleotide-binding domain in facilitating indirect drug efflux in ABCC1. The identified key mutations in endometrial cancer and mapped common mutations present across different types of cancers in ABCB1, ABCC1, and ABCG2 will facilitate the design and discovery of inhibitors targeting unexplored structural regions of these transporters and re-engineering of these transporters to tackle chemoresistance.

## 1 Introduction

Despite tremendous advancements in the treatment of clinical cancer via chemotherapy, radiotherapy, immunotherapy, etc., the development of therapy resistance remains a major challenge and concern for clinicians and researchers alike. The most common mechanisms of resistance include drug efflux, DNA repair, alteration of drug targets, inactivation or sequestration of drug candidates, apoptosis blockage, and genetic/epigenetic alterations across a wide spectrum of anticancer agents (Sajid et al., 2023). Resistance toward anticancer drugs can be classified as either intrinsic or extrinsic, implying that the factors facilitating resistance in cells were present before or after drugs were administered. Of these resistance mechanisms, the ability of cancer cells to identify and selectively efflux a wide spectrum of mechanistically and structurally unrelated drugs is conferred by ATP-binding cassette (ABC) transporters (Juan-Carlos et al., 2021), which can occur due to either intrinsic or extrinsic factors of cancer cells.

ABC transporters are a superfamily of integral membrane proteins with a tissue-specific expression and are responsible for the transfer of many biological substrates across the cell membrane (Robey et al., 2018). In addition to niche biological functions such as export of metabolic end-products and cholesterol in the liver (Wlcek and Stieger, 2014), ABC transporters are involved in efflux of a wide range of chemotherapeutic agents. Consequently, this has serious clinical implications on therapeutic success in patients undergoing chemotherapy.

ABC transporters mainly consist of four domains—two nucleotide-binding domains (NBDs) and two transmembrane domains (TMDs). It can act as both an exporter and importer or may not participate in any exchange at all depending on its location in the body. Usually, ATP or nucleotide binding to NBD causes conformational changes in such a manner that the substrate molecule is captured in its cavity and released on the other side. (Rees et al., 2009; Vasiliou et al., 2009; Tarling et al., 2013; Alam et al., 2019; Di et al., 2022). Of the 48 classified ABC transporters, the three most widely studied and characterized efflux glycoproteins, namely, P-glycoprotein (also known as Pgp or ABCB1), multidrug resistance protein 1 (also known as MRP1 or ABCC1), and breast cancer resistance protein (BCRP) (also known as BCRP or ABCG2), have significant roles in causing multidrug resistance in cancer. Although each of the transporters has been extensively studied and characterized individually, there are gaps in the understanding and comparative analysis of clinically significant mutations at the structural level and its possible implications in chemoresistance.

The NBD regions of ABC transporters are the motor domains where ATP and nucleotide binding occurs. It has already been studied that the ATP-binding regions of these ABC transporters are highly conserved. They consist of several conserved motifs that are involved in ATP binding, ATP hydrolysis, and in facilitating important interfaces in active ABC transporters (Walker et al., 1982; Jones and George, 2004; Hollenstein et al., 2007).

These motifs include the “Walker A motif” also known as p-loop (represented by G-X-X-G-X-G-K-S/T, where X represents any amino acid and common among nucleotide-binding proteins), the core fold of which is characterized by centrally placed parallel beta-sheets flanked by helices, and this motif is responsible for the binding of nucleotides and is essential for ATP hydrolysis; the “LSGGQ motif,” also known to be the “C-loop” or “signature motif” for ABC transporters, interacts with the nucleotide in the ATP-bound state and is essential in ATP hydrolysis as well along with the p-loop; the “Walker-B motif” (represented by h-h-h-h-D, where h signifies hydrophobic residues) offers a conserved glutamate residue that is responsible for the nucleophilic attack on ATP using a water molecule; the “A-loop” consisting of stacked aromatic residues is hypothesized to form a p–p interaction with the bound ATP’s adenine ring; the “Q-loop” is hypothesized to detect the g-phosphate moiety and hence has a role in the contact interface with the transmembrane domain; the “D-loop” is thought to be involved in an interaction between the two NBD regions; and the “switch motif” containing a histidine is said to be involved in a catalytic reaction that takes place in the NBD (Oldham et al., 2008; Zolnerciks et al., 2011; Wilkens, 2015).

Since the NBDs are arranged in a head-to-toe manner, the two nucleotide molecules bound in the NBD region tend to interact with Walker A and Walker B motifs from one NBD and the LSGGQ motif from the other NBD. This arrangement leads to the formation of two ATP-binding sites between Walker A of one NBD and the LSGGQ motif of the other, proving cooperativity in ATP binding and its catalytic activity. In the bound state, two ATP molecules are sandwiched at these interfaces (Walker et al., 1982; Hollenstein et al., 2007).

ABC transporters play a significant role in reproduction. They modulate steroidogenesis, fertilization, fetus implantation, and play a crucial role as a barrier during pregnancy to protect the fetus against the onslaught of harmful xenobiotics, drugs, and environmental toxins (Bloise et al., 2015). Endometrial tissue is known to express all three major ABC transporters—ABCB1, ABCC1, and ABCG2, due to its direct involvement during peri-implantation leading up to placental/fetal development. Hence, in case of endometrial uterine cancer, intrinsic overexpression of all three ABC transporters interferes throughout single-/combination chemotherapy (Arain et al., 2021; Takagi et al., 2023). The response rate in chemotherapy-naïve endometrial carcinoma patients to single-agent chemotherapy (either of doxorubicin, paclitaxel, cisplatin, or carboplatin) is not good and is reportedly below 40% (Moxley and McMeekin, 2010). Furthermore, in case of relapse, there is limited, if any, established second-line chemotherapy/agent to administer to women with an MDR endometrial cancer profile.

**FIGURE 1:**
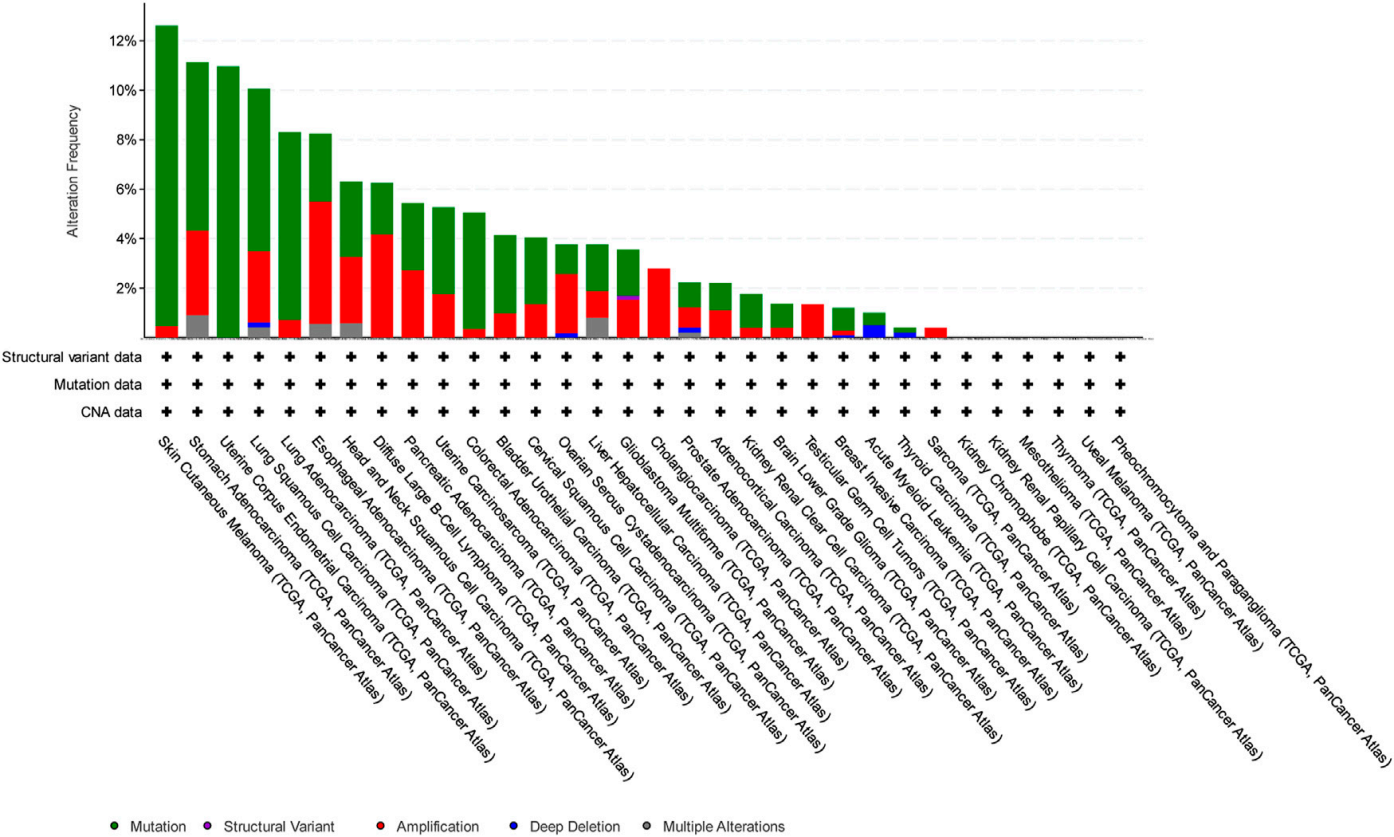
Overview of the frequency of clinical variations observed in ABCB1 in different cancers. The uterine corpus endometrial carcinoma was among the top two cancers with respect to mutation frequency for ABCB1.

**FIGURE 2:**
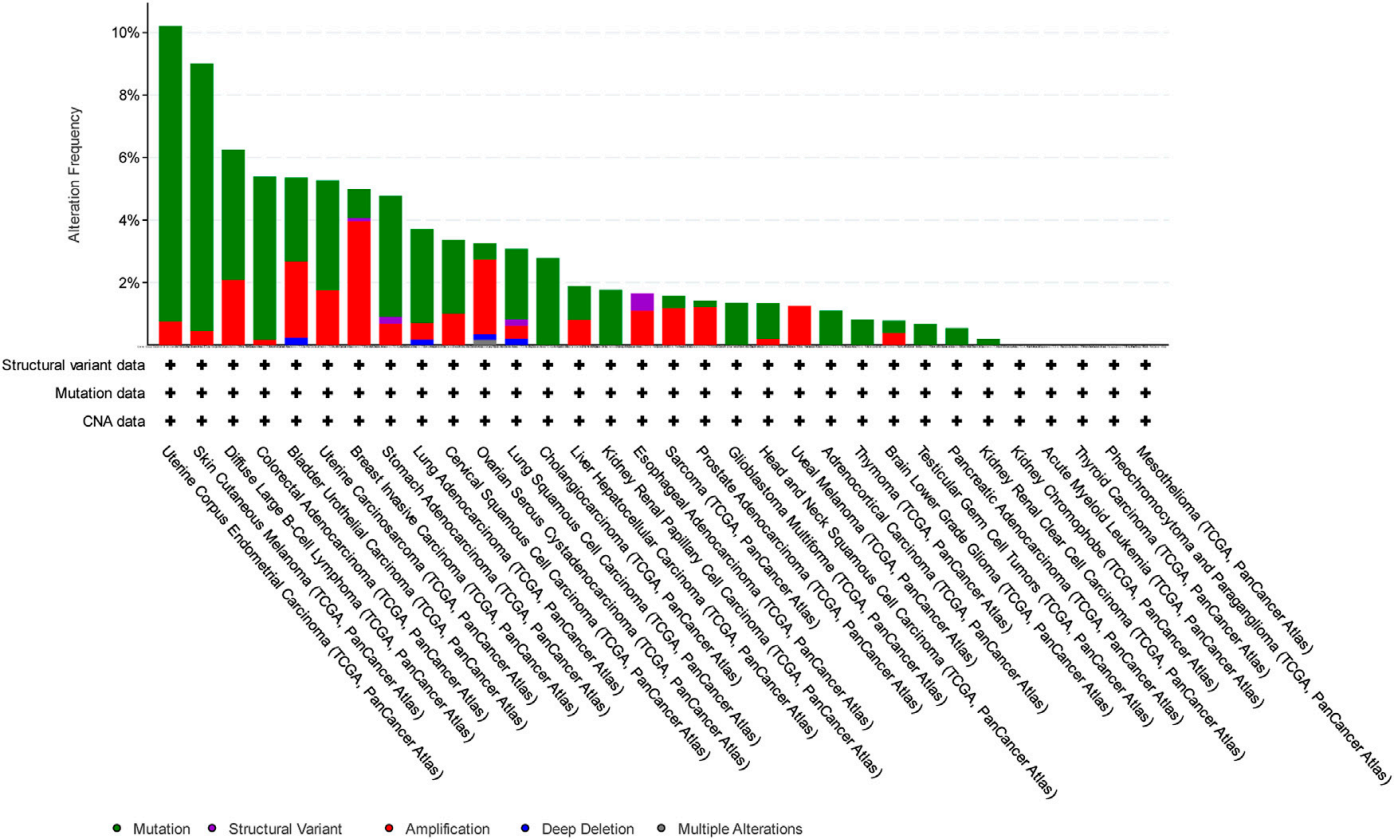
Overview of the frequency of clinical variations observed in ABCC1 in different cancers. The uterine corpus endometrial carcinoma shows the highest mutation frequency for ABCC1.

**FIGURE 3:**
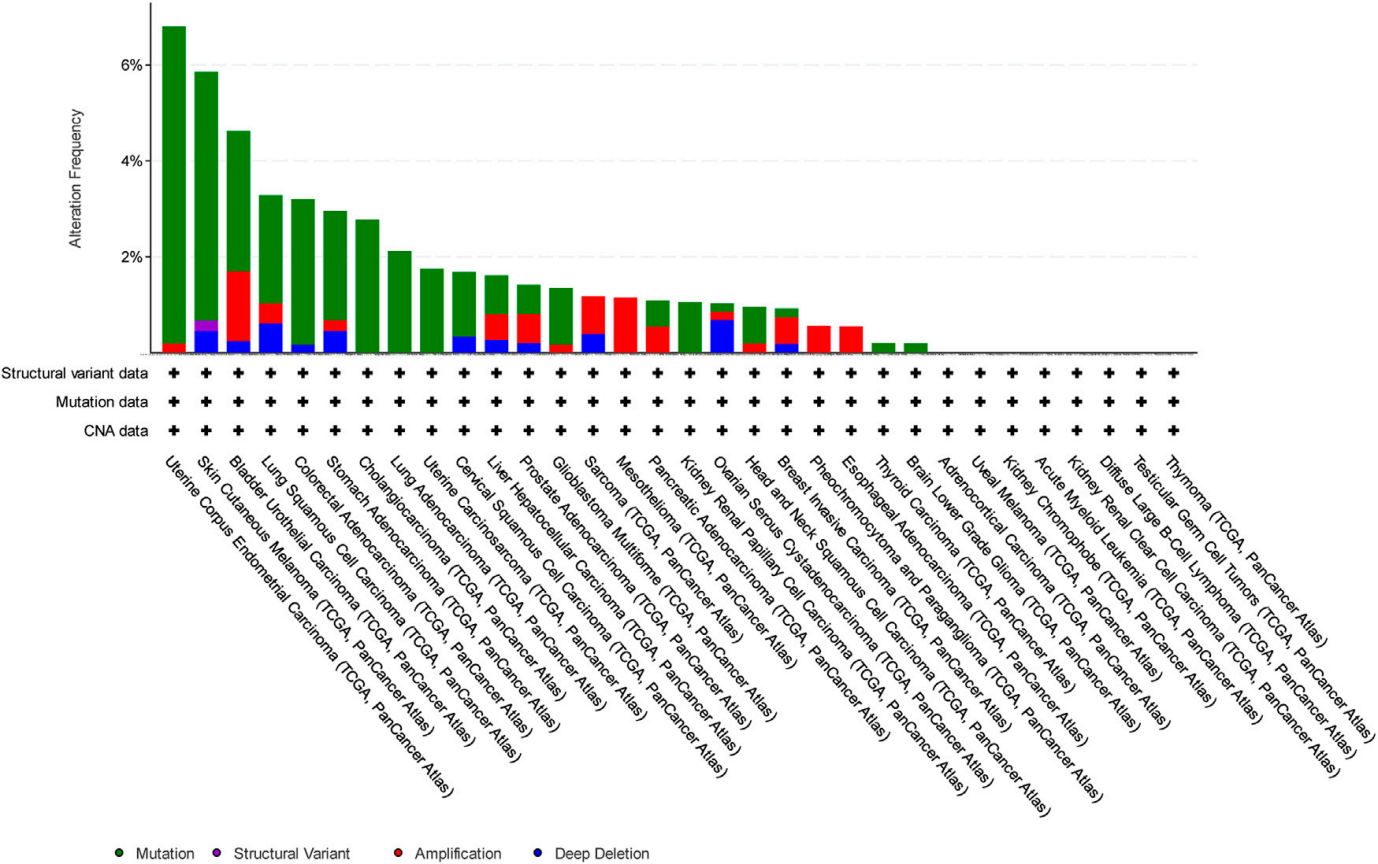
Overview of the frequency of clinical variations observed in ABCG2 in different cancers. The uterine corpus endometrial carcinoma shows the highest mutation frequency for ABCG2.

In this study, we have focused on endometrial cancer due to the abundance of mutation data, which were relatively higher than those of other cancer types, as shown in Figure 1, Figure 2, and Figure 3 for ABCG2, ABCB1, and ABCC1, respectively, taken from cBioPortal for cancer genomics (Gao et al., 2013). The data are part of TCGA PanCancer data for uterine corpus endometrial carcinoma. Out of a total of 529 cases, the ABCB1, ABCC1, and ABCG2 transporters were found to be mutated in 10.96%, 9.45%, and 6.62% of the cases, respectively (Berger et al., 2018; Hoadley et al., 2018).

Mitoxantrone is a topoisomerase type IIα inhibitor, extensively used for the treatment of acute myeloid leukemia (AML), acute lymphoblastic leukemia (ALL), and advanced prostate cancer, besides other conditions. The mechanism of action in cancer cells involves disruption of DNA synthesis and prevention of re-ligation of broken DNA strands, thereby impairing DNA repair function. For this work, we have taken mitoxantrone as the model substrate as it is known to confer resistance and lead to upregulation of all the three ABC transporters of interest: ABCG2, ABCB1, and ABCC1 (Nieth and Lage, 2005). Stefan et al. curated a list of functional tracers based on extensive literature search of experimental data. Of the various molecules, only Calcein AM, Rhodamine-123, and mitoxantrone were used to evaluate potential inhibitors against all three transporters—ABCB1, ABCC1, and ABCG2. Out of these compounds, since only mitoxantrone had both functions—as a tracer and chemotherapeutic agent, therefore we decided to perform molecular docking with mitoxantrone for all the three transporters (Stefan et al., 2022). Mutagenesis studies in the drug-binding pockets across ABC-transporter variants have demonstrated the presence of multiple overlapping sites where drug or inhibitors can bind, referred to as polyspecificity (Sajid et al., 2023). This leads to multidrug resistance, and hence, it is imperative to evaluate the common binding points, which can aid in better designing of therapeutics (small-molecule inhibitors/peptides, etc.) which can evade efflux by ABC transporters.

## 2 Materials and methods

### 2.1 Data collection and pre-processing

Mutation data for this study were collected from cBioPortal (Cerami et al., 2012) developed at Memorial Sloan Kettering Cancer Center (MSKCC) which hosts data from The Cancer Genome Atlas (TCGA) (Weinstein et al., 2013), National Institute of Health (NIH). TCGA catalogs the genetic mutations responsible for different cancers using genome sequencing. TCGA was supervised by the National Cancer Institute’s Center for Cancer Genomics and the National Human Genome Research Institute. Out of all the listed mutations in ABCB1, ABCC1, and ABCG2 for endometrial cancer, only mutations supported by at least two mutation functional impact prediction methods (Mutation Assessor, SIFT and PolyPhen-2) for their deleterious or damaging impact were selected (Ng and Henikoff, 2001; Adzhubei et al., 2010; Reva et al., 2011) (Supplementary Material).

### 2.2 Molecular docking and visualization

The three-dimensional structures for the ABCB1, ABCC1, and ABCG2 transporters were taken from the Research Collaboratory for Structural Bioinformatics (RCSB) Protein Data Bank. (Gao et al., 2013; Kowal et al., 2021; de Bruijn et al., 2023). PDB files for docking were taken from the RCSB with ID 7NFD(31) for ABCG2, 7A69 (Nosol et al., 2020) for ABCB1, and 6UY0 (Wang et al., 2020) for ABCC1. The mitoxantrone structure was taken from ChEMBL (Gaulton et al., 2012; Zdrazil et al., 2024). The list of residues that are known to interact with mitoxantrone or other drug molecules for ABCG2, ABCB1, and ABCC1 was used during molecular docking. Molecular docking was performed between ABC transporters and mitoxantrone individually using LeDock (Liu and Xu, 2019), and the complex structures were visualized for interactions in the 2D mode using LigPlus (Laskowski and Swindells, 2011). Three-dimensional visualization was performed using PyMOL (PyMOL, 2015; Yuan et al., 2017).

### 2.3 Classification of key residues

All the mutations that were considered for the analysis based on their possible functional impact in ABCB1, ABCC1, and ABCG2 were further classified based on their location in the three-dimensional structure as follows: in the drug binding/transport channel, at the entry or exit, the membrane-facing regions, and the nucleotide- or ATP-binding regions. We have also taken inputs from the annotations provided at the cBioPortal for this purpose (Gao et al., 2013).

## 3 Results

Mutation data collected from the cBioPortal contained a total of 891 mutations across three ABC transporter sub-families, 480 in ABCB1, 294 in ABCC1, and 117 in ABCG2. Out of these, mutations observed in uterine corpus endometrial carcinoma were taken into account for further analysis, which narrowed down to a total of 248 mutations: 118 in ABCB1, 82 in ABCC1, and 48 in ABCG2.

### 3.1 Key mutations involved in drug binding

After review of the literature, a list of residues that have been shown to interact with the drugs or substrate molecules was curated for all the three ABC transporters. We found a total of 22 residues in ABCB1, 27 residues in ABCC1, and 28 residues in ABCG2, as shown in the first column of Table 1, Table 2, and Table 3, respectively. These residues were then compared with the TCGA mutation data for any potential matches, wherein we observed six matches in ABCB1 (W232L, A987T, X235_splice, G872V, A230T, and S993A), six matches in ABCC1 (M1092V, G1319R, A438V, V1325M, C1208Y, and V776M), and three matches in ABCG2 (L405M, E446N, and L595I), as shown in the second column of Tables 1–3, respectively. These matches were significant for determining the location of our grid box for molecular docking.

**TABLE 1:** ABCB1 mutation data. The first column represents residues known to interact with drug molecules from the literature survey; the second column represents the matches between known residues and TCGA dataset/mutations; and the third column represents the residues found to be interacting after docking of mitoxantrone to ABCB1 using LeDock. The highlighted residues are the novel residues observed to be interacting with mitoxantrone.

Residues known to interact with drug molecules	Matching mutations from TCGA	Residues involved in binding with mitoxantrone
Q725	W232L	M192
F728	A987T	Q195
A871	X235_splice	W232
E875	G872V	F343
L879	A230T	Q347
Q946	S993A	A871
M949		G872
Y950		E875
Y953		M878
F978		T945
S979		Q946
V981		M949
V982		M986
F983		Q990
M986		
A987		
Q990		
V991		
F994		
Y1044		
R1047		
Q1118		

**TABLE 2:** ABCC1 mutation data. The first column represents residues known to interact with drug molecules from the literature survey; the second column represents the matches between known residues and TCGA dataset; and the third column represents the residues found to be interacting after docking of mitoxantrone to ABCC1 using LeDock. The highlighted residues are the novel residues observed to be interacting with mitoxantrone.

Residues known to interact with drug molecules	Matching mutations from TCGA	Residues involved in binding with mitoxantrone
K332	M1093V	F389
H335	G1320R	Q432
L381	A438V	M435
H382	V1326M	D436
F385	C1209Y	T439
R433	V776M	Y440
D436		S1084
Y440		M1085
T550		Q1088
W553		M1092
F594		
M601		
G681		
S769		
Q1088		
M1092		
S1096		
Y1188		
R1196		
E1203		
Y1242		
N1244		
W1245		
R1248		
M1249		
G1329		
S1430		

**TABLE 3:** ABCG2 mutation data. The first column represents residues known to interact with drug molecules from the literature survey; the second column represents the matches between known residues and TCGA dataset; and the third column represents the residues found to be interacting after docking of mitoxantrone to ABCG2 using LeDock. The highlighted residues are the novel residues observed to be interacting with mitoxantrone.

Residues known to interact with drug molecules	Matching mutations from TCGA	Residues involved in binding with mitoxantrone
Q141	L405M	S420
Q181	E446N	Q424
F182	L595I	A427A
R184		G428A
V401		F432
L405		T435
F431		N436
F432		F439
T435		S440
N436		S443
F439		T542
S440		V546
V442		M549
E446		L554
R482		L555B
V534		A606
T538		T607
L539		Y613B
T542		K616B
I543		
V546		
M549		
L554		
L555		
C592		
N596		
C603		
C608		

The PDB structure taken for ABCG2, 7NFD had already mitoxantrone bound to it, making it a reference for our results. The comparison with complex structures generated by LeDock (Liu and Xu, 2019) has shown similar amino acid residues taking part in the molecular interaction. This result allowed us to use the same docking methodology to build all the complex structures.

**FIGURE 4:**
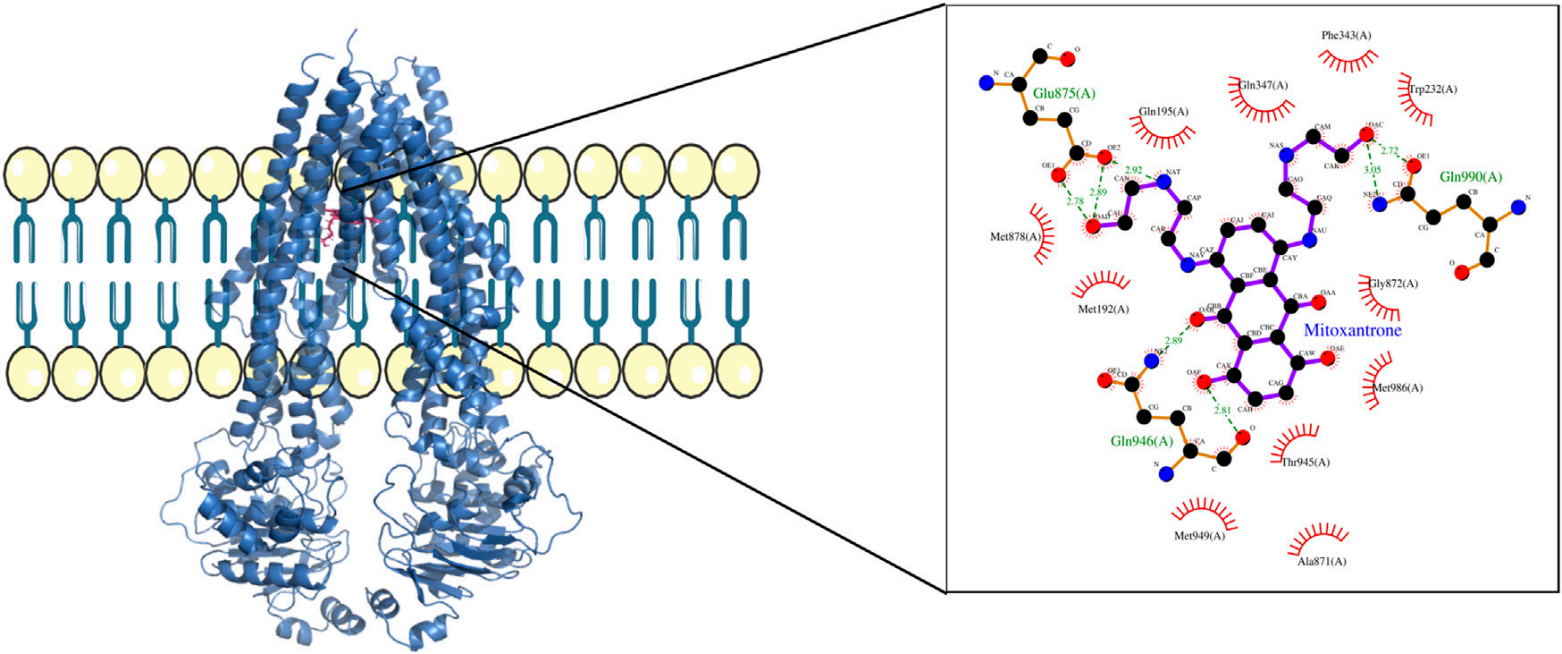
ABCB1–mitoxantrone complex structure highlighting the residues in ABCB1 interacting with mitoxantrone. The molecular interaction representation on the right shows the non-covalent interactions between the key amino acid residues in ABCB1 and mitoxantrone.

**FIGURE 5:**
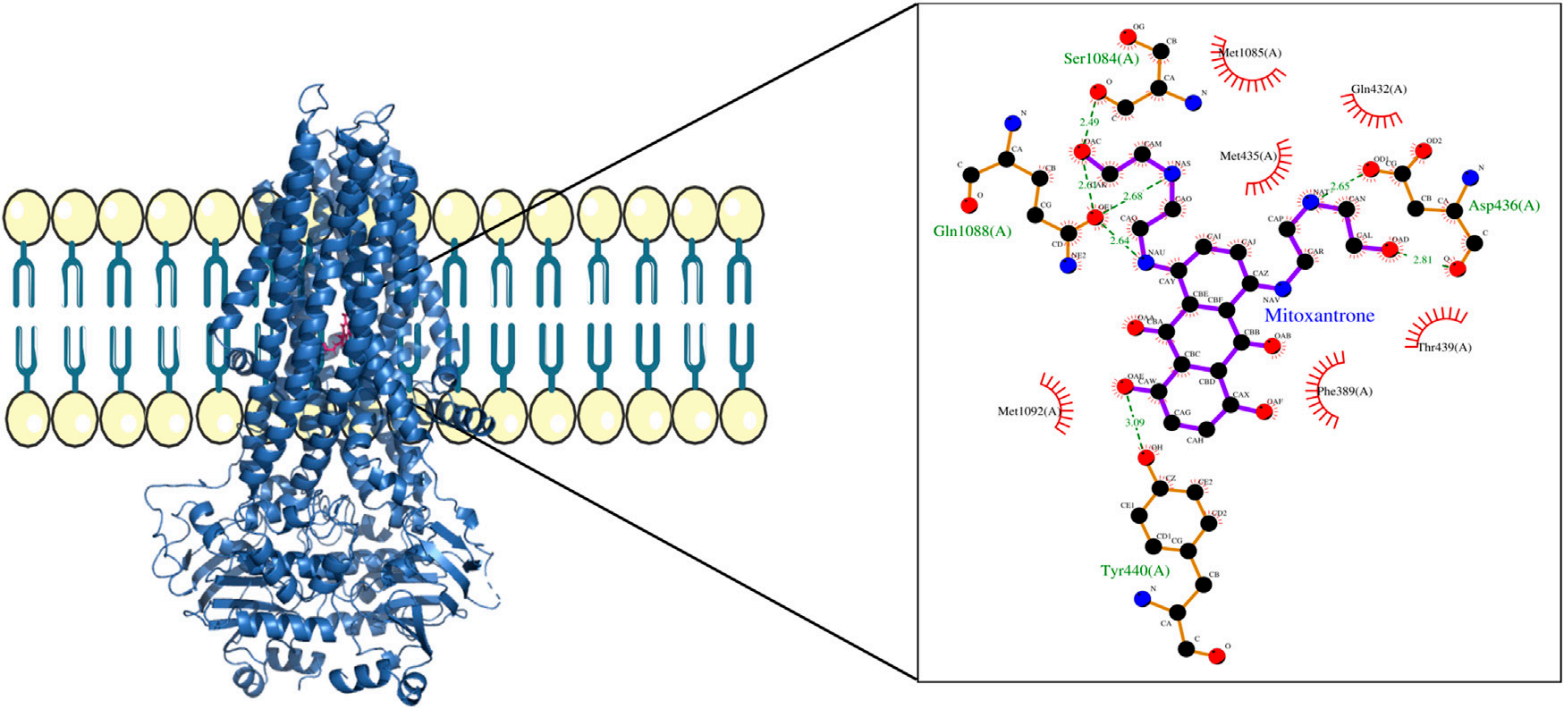
ABCC1–mitoxantrone complex structure highlighting the residues in ABCC1 interacting with mitoxantrone. The molecular interaction representation on the right shows the non-covalent interactions between the key amino acid residues in ABCC1 and mitoxantrone.

**FIGURE 6:**
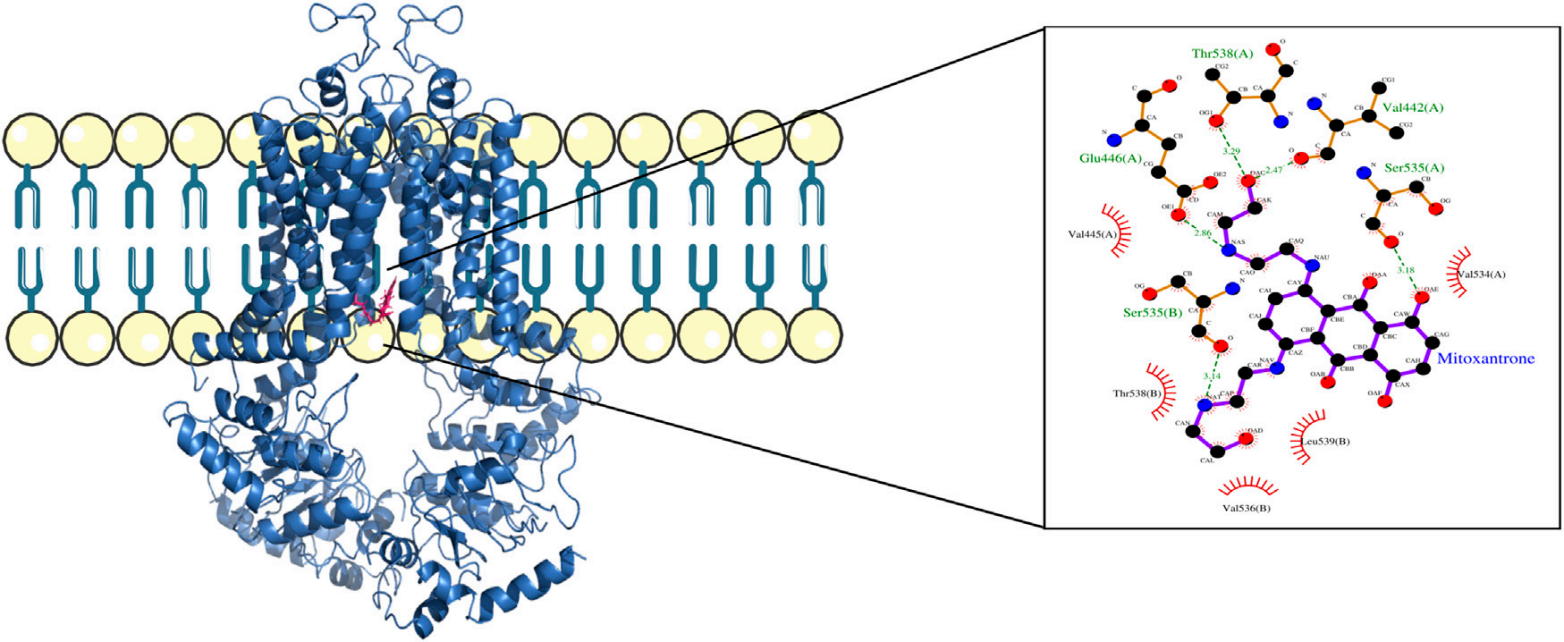
ABCG2–mitoxantrone complex structure highlighting the residues in ABCG2 interacting with mitoxantrone. The molecular interaction representation on the right shows the non-covalent interactions between the key amino acid residues in ABCG2 and mitoxantrone.

For structural level interaction studies between ABC transporters and substrate/drug molecules, molecular docking was performed for the selected PDB structures of ABCB1, ABCC1, ABCG2, and mitoxantrone using LeDock (Liu and Xu, 2019). After molecular docking, the complexes were then analyzed for visualizing molecular interactions between mitoxantrone and amino acid residues using a LigPlot (Laskowski and Swindells, 2011)-based two-dimensional interaction diagram (Figure 4; Figure 5; Figure 6).

In Figure 4, the structure of the complex between ABCB1 and mitoxantrone can be seen. Novel residues found to be interacting with mitoxantrone were M192, Q195, F343, Q347, M878, and T945. All the interacting residues are also listed in the third column of Table 1, and the interactions can be seen in a two-dimensional representation on the right side in Figure 4. Similarly, in Figure 5, the structure of the complex between ABCC1 and mitoxantrone can be seen. Novel residues found to be interacting with mitoxantrone were F389, Q432, M435, T439, S1084, and M1085. All the interacting residues are also listed in the third column of Table 2, and the interactions can be seen in the two-dimensional representation on the right side in Figure 5. Figure 6 shows the structure of the complex formed between ABCG2 and mitoxantrone. The two-dimensional visualization of interactions can be seen on the right side in Figure 6. Out of all the interactions observed, many amino acid residues were similar to the residues already reported to interact with the ABC transporters. However, few novel residues S420, Q424, A427, G428, S443, A606, T607, Y613, and K616 were also shown to interact with mitoxantrone, and these residues along with all the interacting residues that were observed are also listed in the third column of Table 3.

The description of mutations in ABCB1, ABCC1, and ABCG2 that are also present in other different cancers (such as cancers of the brain, lung, colon, prostate, and kidney) in addition to endometrial cancer is provided in Table 4.

**TABLE 4:** Mutations in ABCB1 (nine mutations) are highlighted in pink, ABCC1 (four mutations) are highlighted in green, and ABCG2 (one mutation) is highlighted in purple. These functionally important mutations were present in other different cancers in addition to endometrial cancer. The cancer types for each mutation are listed on the right most column of the table.

Gene	Mutation	Location of mutation in ABC transporters	Cancer type
**ABCB1**	R958W	Exit site of the channel	Uterine carcinosarcoma/uterine malignant mixed Mullerian tumor
			Glioblastoma multiforme
	V974F	Exit site of the channel	Uterine endometrioid carcinoma
			Lung adenocarcinoma
	A819T	Drug transport channel	Uterine endometrioid carcinoma
			Colon adenocarcinoma
	R789Q	Drug transport channel	Uterine carcinosarcoma/uterine malignant mixed Mullerian tumor
			Cutaneous melanoma
	R41H	Membrane-facing region	Uterine endometrioid carcinoma
			Mucinous adenocarcinoma of the colon and rectum
			Colon adenocarcinoma
	R489C	Membrane-facing region	Glioblastoma multiforme
			Uterine endometrioid carcinoma
			Cutaneous melanoma
	K30N	NBD/ATP-binding domain	Uterine endometrioid carcinoma
			Mucinous adenocarcinoma of the colon and rectum
	G449R	NBD/ATP-binding domain	Uterine endometrioid carcinoma
			Cutaneous melanoma
	R467W	NBD/ATP-binding domain	Prostate adenocarcinoma
			Uterine endometrioid carcinoma
			Uterine serous carcinoma/uterine papillary serous carcinoma
			Signet ring cell carcinoma of the stomach
			Mucinous adenocarcinoma of the colon and rectum
**ABCC1**	D355Y	Exit site of the channel	Uterine endometrioid carcinoma
			Cutaneous melanoma
	R501Q	Drug transport channel	Renal clear cell carcinoma
			Uterine endometrioid carcinoma
			Colon adenocarcinoma
	E1144K	Drug transport channel	Prostate adenocarcinoma
			Uterine endometrioid carcinoma
			Head and neck squamous cell carcinoma
	R758	NBD/ATP-binding domain	Uterine endometrioid carcinoma
			Cutaneous melanoma
**ABCG2**	K653E	Membrane-facing region	Uterine endometrioid carcinoma
			Colon adenocarcinoma

The highlighted mutations in ABCB1, ABCC1, and ABCG2 (Tables 4, 5, and 6) would be highly important from the point of view of functionality of these transporters. Since these specific mutations are reported in more than one cancer type, their contribution to chemoresistance in these transporters holds more clinical relevance and therapeutic significance.

### 3.2 Functional impact of mutations present in ABCB1

**FIGURE 7:**
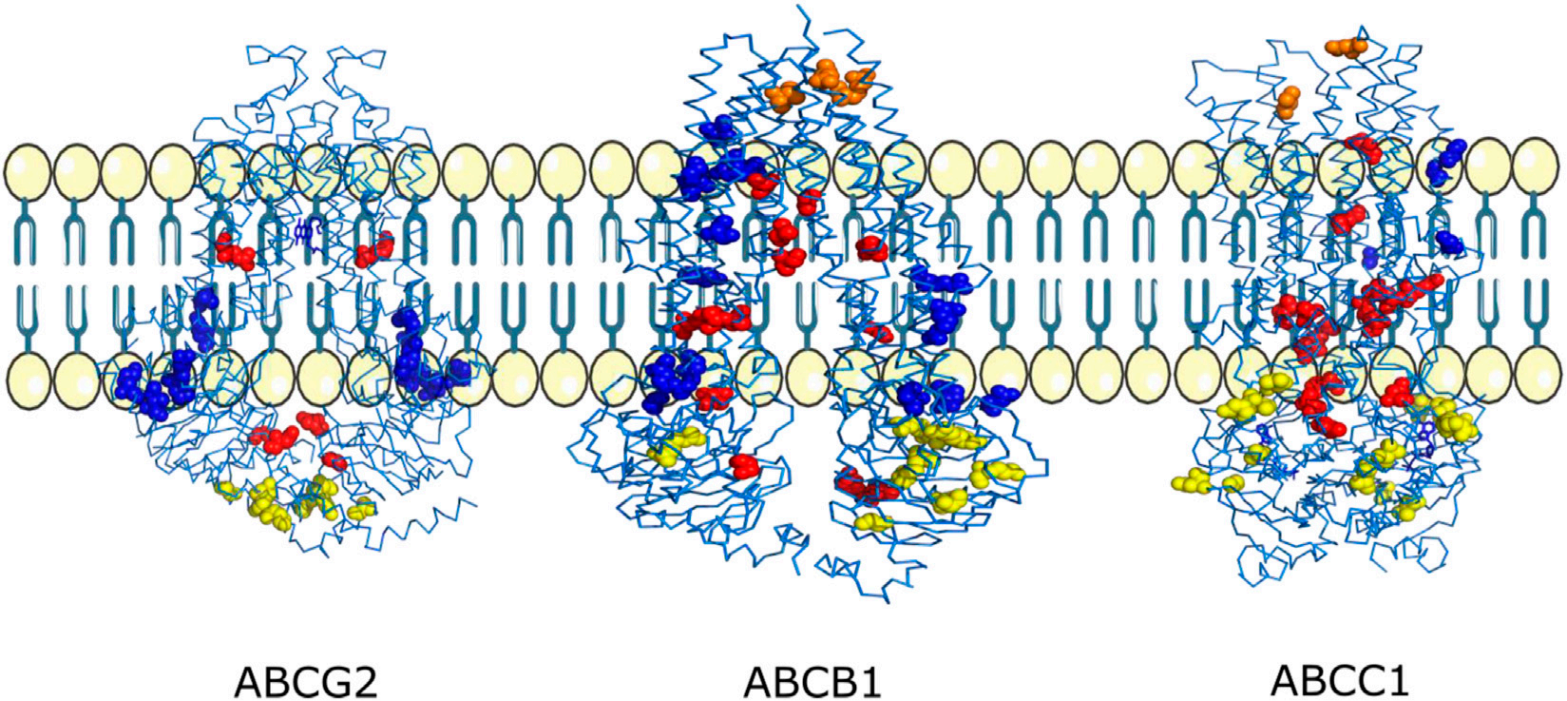
Mutations mapped onto 3D structures of the ABCG2, ABCB1, and ABCC1 transporters. Mutations observed near the exit of the drug transport channel are shown in orange, mutations observed inside the drug transport channel are shown in red, mutations observed on the membrane-facing regions are shown in blue, and mutations observed at the ATP/nucleotide-binding regions are shown in yellow.

We found that three residues D77, V974, and R958 found on the exit site of the channel were shown to be mutated into hydrophobic residues. All these mutations will lead to the increase of drug efflux because it is known that ABCB1 majorly transports hydrophobic and amphipathic compounds, and all these mutations will enhance ABCB1’s capability to transport such compounds that can possibly lead to chemoresistance (Figure 7; Table 5).

**TABLE 5:** Key mutations mapped to the important structural regions of the ABCB1. The highlighted mutations are also present in other cancer(s) in addition to endometrial uterine carcinoma. Mutations shown in bold were reported to be important in drug response as per ClinVar (Landrum et al., 2020).

Exit site of the channel	Drug transport channel	Membrane-facing regions	NBD/ATP-binding domain
D77Y	D188G	R41H	K30N
R958W	A230T	Q158H	V437A
V974F	S237Y	G269E	G449R
	A246V	R276M	R467W
	D562G	S298Y	V472M
	E566K	R489C	**R543H**
	**R789Q**	R489H	R547H
	V801A	N494H	K550N
	A819T	F697C	T582A
	G872V	G763V	G604E
	G892C	S766Y	R1188C
	A987T	Q799H	
	S1177F	N842H	
		L843I	
		I848S	
		R929I	

Mutations A987T, G872V, A230T, S237Y, and D188G are located close to the binding site of the drug molecule inside the channel, and mutations A819T, A246V, and R789Q indicate that it will lead to an increase in the amphiphilic nature of the channel that can lead to binding of diverse drug compounds and seems important in explaining the polyspecificity in ABCB1 important for chemoresistance. The R789Q mutation was also shown to be important in the drug response as per ClinVar (Landrum et al., 2020). Mutations S1177F, V801A, and G892C are present close to the entry and intramembrane region, with the closest being S1177F. S1177F and V801A can lead to an increase in hydrophobicity and entry volume of the channel, which is important in entry of the drug from the intracellular environment, making it more efficient, which in turn helps in chemoresistance (Figure 7; Table 5).

Mutations E566K and D562G are very closely located on the entry from the intracellular side. Common drugs like paclitaxel and cyclosporine A (known to have an effect in ABCB1) contains hydroxyl and keto group and aspartic acid residues, respectively, which are negatively charged; therefore, due to mutations from glutamate to lysine, they can effectively be impacted via charged mutated and hydrogen bonding interactions that can lead to better drug efflux. The other mutation D562G can be important in increasing the volume of the intracellular entry side, important for accessing the drug-binding channel (Figure 7; Table 5) (Sissung et al., 2006; Chang et al., 2009; García et al., 2013; Zhang et al., 2013; Gao et al., 2014; Damont et al., 2016; Vallo et al., 2017; van Eerden et al., 2021).

According to the hydrophobic match/mismatch theory, transmembrane proteins avoid unfavorable exposure of the hydrophobic surface present on the transmembrane region of the protein to a hydrophilic environment present on the lipid bilayer surfaces. Biological membranes tend to adapt to such mismatch by expanding and shrinking. Lipid bilayers get adjusted elastically to the hydrophobic length of the transmembrane helices where a decreased hydrophobic transmembrane length would lead to shrinkage of the lipid bilayer along the transmembrane region, reducing the curvature of the overall membrane, whereas increased hydrophobic transmembrane length would lead to elongation of the bilipid layer along the transmembrane region, increasing the curvature of the overall membrane. We discussed the possible effects of this phenomenon with respect to mutations in the ABCB1, ABCC1, and ABCG2 transporters (Weiss et al., 2003; Janmey and Kinnunen, 2006; Soubias et al., 2008; de Jesus and Allen, 2013; Engberg et al., 2022).

With mutations I848S, L843I, and N842H, here threonine to serine will change from non-polar to polar residues, leucine to isoleucine will change from large hydrophobic residues to smaller hydrophobic residues, and asparagine to histidine will change from polar to positively charged amino acids. All these three mutations can lead to a favorable interaction with the non-polar membrane component and the polar component of the cholesterol molecule. For example, asparagine to histidine mutation will introduce an imidazole side chain which can interact favorably with the polar part cholesterol and non-polar part of the phospholipid membrane. Overall, these mutations will help in increasing the fluidity of the cell membrane.

Other mutations like S766Y, G763V, and S298Y would lead to an increase in the non-polar interaction between these residues and the non-polar part of the membrane. However, the hydroxyl group present in tyrosine can also interact favorably with cholesterol molecules. These mutations can lead to a probable increase in the rigidity of the membrane. With mutations R276M and G269E, arginine to methionine will change from charged to non-polar residues, which will lead to favorable interactions for non-polar membrane components. With mutation G269E, glycine to glutamic acid will lead to favorable interactions with cholesterol molecules present on the membrane, important for maintaining the fluidity and stability of the membrane. With mutation Q799H, glutamine to histidine will favor interactions with cholesterol molecules, leading to an increase in fluidity and stability of the membrane.

Q158H, R489H/C, and N494H mutations from polar to positively charged residues like Q158H and N494H can lead to favorable interactions with cholesterol and other polar molecules present on the membrane, important for maintaining the membrane fluidity and stability. On the other hand, R489H and R41H mutations will lead to a favorable interaction with cholesterol and polar molecules, and also an imidazole ring can interact favorably with the non-polar components of cholesterol and phospholipid membrane, signifying a dual role in histidine. Mutation F697C which is facing outwards toward the membrane may favorably interact with cholesterol and polar molecules required for maintaining the fluidity of the cell membrane and mutation R929I with hydrophobic nature would have favorable interactions with the non-polar component of the membrane and cholesterol.

The mutations L843I, S766Y, G763V, S298Y, R489H/C, F697C, and R929I present in the transmembrane region of ABCB1 would result in a change in hydrophobic interactions, which can modulate the hydrophobic mismatch. This would lead to a change in membrane curvature and/or fluidity affecting the ABCB1 conformational dynamics important for the drug transport and efflux.

In the ATP-binding site 1, we observed mutations V437A and R467W, both of which will lead to an enhanced ATP binding or release through the Walker motif A and B. Since V437A will lead to decrease in the side chain volume, it will allow more conformational freedom for the adjacent residues T435 of the Walker motif A. The R467W mutation will lead to an increase in the volume, and due to its hydrophobic nature, it would lead to reorientation of the R441 residue located opposite to that, which can restrict the conformational freedom of the Walker A motif important in ATP binding and hydrolysis (Loo et al., 2002; Orelle et al., 2003; Rai et al., 2006; Szöllősi et al., 2020).

V472M and G604E mutation will lead to an increase in the side chain volume, leading to the restriction of movement of the Walker A motif, thus favoring the ATP bound conformation which may have importance in chemoresistance. G449R mutation to the positively charged residue can form favorable interactions with adjacent negatively charged glutamic acid residues (E448), which will in turn lead to stabilization of an adjacent long loop which is directly connected to the Walker A motif. Mutations R543H and R547H which are located adjacent to each other upon mutation to histidine will lead to overall greater conformational freedom, allowing an optimal interaction between Walker A motif and Q loop for better ATP binding and hydrolysis. The R543H mutation was also shown to be important in the drug response as per ClinVar (Landrum et al., 2020). Mutations K550N and T582A will lead to a decrease in the overall volume of the side chain of amino acid residues, allowing more conformational freedom for the interaction between Walker A motif and ATP for optimal binding and hydrolysis.

Mutation R1188C located on the ATP-binding domain 2 is present in the vicinity of Q loop and Walker B motif; therefore, the mutation from R to C will allow more conformational freedom for better interaction and thus better binding with ATP molecules. The G604E mutation, which is located close to the cystine C431 residue of the Walker A motif, may lead to formation of polar interactions with C431 and would restrict the movement of the Walker A motif that may stabilize the ATP bound form of the ATP-binding domain. Mutations in NBD regions can be targeted to inhibit or reduce the ATP hydrolysis which is important for drug transport and hence chemoresistance.

### 3.3 Functional impact of mutations present in ABCC1

D355Y mutation present on the exit site of this transporter may facilitate efficient efflux of diverse drug compounds since the side chain of tyrosine containing the hydroxyl group can act as a hydrogen bond donor or acceptor. Furthermore, the aromatic side chain of tyrosine can facilitate a hydrophobic interaction with diverse drug compounds. V584A mutation which is present on the opposite site of the D335 mutation will lead to increase in exit cavity volume, leading to efficient drug efflux (Figure 7; Table 6).

**TABLE 6:** Key mutations mapped to the important structural regions of the ABCC1. The highlighted mutations are also present in other cancer(s) in addition to endometrial uterine carcinoma.

Exit site of the channel	Drug transport channel	Membrane-facing regions	NBD/ATP-binding domain
D355Y	A398T	G226W	T21M
V584A	G421R	G971R	G78R
	M495T	G971R	Y91C
	M495V	P1121S	L153P
	R501Q	G1123D	R171H
	E507K		E298K
	E507K		V409A
	M1093V		L625M
	R1142C		E626G
	E1144K		N648K
	R1148H		A690S
	Q1239H		A690T
			N719T
			R758W
			V776M
			V776M
			L778M
			G844S
			G1320R
			V1326M
			E1345K
			A1359T
			I1361T
			V1162G

Mutation Q1239H located close to the binding site will allow both the charge-mediated and non-polar interaction with diverse drug compounds. Since this mutation is located just above the Y1242 residue of the ABCC1 binding pocket, it can also stabilize the binding pocket through pi–pi stacking between the histidine and tyrosine residues. Thus overall, this mutation may enhance the efflux of diverse drug compounds. Mutation M1093V takes place in the H-pocket which comprises W1245, Y1242, M1093, T550, and W553. Upon mutation to valine, there will be a reduction in the site chain volume; therefore, the H-pocket can accommodate binding of both smaller and larger drug molecules mediated by a hydrophobic interaction (Figure 7; Table 6).

Mutations R1142C present on the channel lining will lead to an increase in the volume of the channel and will also facilitate the transport of diverse amphipathic drug compounds. E1144K mutation, which is located in the vicinity of R1146, K1139, and R1140, will lead to repulsion due to like charges and, thus, may lead to an increase in channel volume and flexibility.

The R1148H mutation is again located on the channel lining. Histidine’s imidazole ring would lead to better non-polar interactions in addition to charge-mediated interactions with diverse amphipathic drug molecules. It would also lead to the reduction in the side chain volume, resulting in a probable increase in channel volume, favoring efficient drug efflux. In residue M495, it is found to be mutated in two different cancer samples to threonine and valine. In both cases, it will lead to a decrease in the volume of the side chain, leading to easy efflux of diverse amphipathic drug molecules. R501Q mutation will lead to a decrease in the side chain volume without losing the capability of forming a polar interaction with drug molecules for efficient efflux. G421R mutation located adjacent to E422 might lead to favorable charge-mediated interaction, leading to the stabilizing of the helix which forms the efflux channel (Figure 7; Table 6).

The A398T mutation is surrounded by residues V253, V256, and L257. So this mutation will change the hydrophobic nature of the side chain to polar and, therefore, may form unfavorable interactions with the surrounding residues while pushing itself toward the drug efflux channel, thus facilitating the interaction with amphipathic drug molecules. E507K mutation is located in close proximity to K503 and is involved in the formation of polar contact. Thus, this mutation leading to a lysine residue in this position pushes this residue toward the entry of the channel, which may be important in regulating the drug axis and efflux.

In the NBD2 region of ABCC1, mutation V1326M will lead to increase in the hydrophobic side chain volume, leading to better packing with the Walker A motif and surrounding hydrophobic residues.

Residues V776 and L778 are sandwiched between LSGGQ and Walker motif B in the NBD1 region. Mutations V776M/M and L778M, where the residues are directly interacting with the LSGGQ motif, upon mutation to methionine, will lead to a better interaction with the LSGGQ motif and the other surrounding residues. G844S, A690S, and A690T are located close to Walker motif A in NBD1. All these mutations will lead to a hydrophobic to polar side chain that will lead to a favorable polar interaction with the charged and polar residue of Walker motif A.

The G971R mutation will lead to formation of polar interactions with cholesterol and other polar molecules present in the phospholipid membrane required for maintaining membrane fluidity. G226W mutation located in the vicinity of two other tryptophan residues W223 and W242 will lead to the formation of strong hydrophobic clusters, thus stabilizing the L_0_ region (205–268) which is known to be essential for folding of ABCC1 (Weinstein et al., 2013). P1121S and G1123D both are located facing the membrane. These mutations will lead to a better interaction with cholesterol and the polar component of the phospholipid membrane, thus providing membrane fluidity required for efficient drug efflux.

We observed a higher number of key mutations in the NBD1 region as compared to the NBD2 region of ABCC1. It is reported that in ABCC1, only the NBD2 region is catalytically active (Sajid et al., 2023). Therefore, a higher number of mutations in NBD1 might be important in retaining the activity of NBD2, which is important for drug transport, while increasing the binding affinity of the NBD1 region through mutations.

### 3.4 Functional impact of mutations present in ABCG2

R482K, D217Y, and G80R mutations are located close to the entry site from the intracellular side of ABCG2. R482K will lead to an increase in channel volume without affecting the formation of a charge-mediated interaction with diverse drug compounds, while mutation of D217Y to tyrosine can facilitate the efflux of diverse amphipathic drug molecules through side-chain flipping mediated by favorable interactions. G80R mutation located to arginine provides a stable interaction with the charged substrate and drug molecule (Figure 7; Table 7).

**TABLE 7:** Key mutations mapped to the important structural regions of the ABCG2. The highlighted mutations are also present in other cancer(s) in addition to endometrial uterine carcinoma.

Drug transport channel	Membrane-facing regions	NBD/ATP-binding domain
G80R	E138K	Y247C
D217Y	L140V	A270T
R482K	K157T	E285K
	I161S	A304D
	L648F	
	K653E	

The E138K mutation would probably make contact with the hydrophilic head of the membrane which is often negatively charged; therefore, this mutation will facilitate favorable charge-mediated interactions. L140V mutation will allow more flexible hydrophobic interactions with the nearby residues such as I161, thereby helping modulate the flexibility of this region. K157T mutation will lead to formation of a better interaction with nearby residues such as T150, N154, and M152, therefore playing a crucial role for enhancing the stability of this whole region. Mutation I161S will lead to the formation of favorable polar interactions with E138 and K157 (Figure 7; Table 7).

Mutation L648F facing toward the membrane region and located in the vicinity of F650 will lead to stabilization of the helix through hydrophobic interactions and can also interact favorably with the non-polar region of the phospholipid membrane. Mutation K653E facing toward the membrane region may interact favorably with the phospholipid membrane and also the polar molecule present in the phospholipid membrane and therefore may be crucial for membrane stability.

In ABCG2, mutations L140V, K157T, and L648F changing to hydrophobic residues may lead to hydrophobic matching by increasing and elongating the bilipid layer across the transmembrane region, contributing in its flexibility and hence important in drug transport and efflux.

The Y247C, A270T, E285K, and A304D mutations are located on the entry region on the intracellular side. Mutations Y247C and A304D, wherein A304D mutation also becomes larger due to an increase in the side chain volume, will allow flexible polar interactions with diverse drug substrates. The A270T mutation will replace a smaller residue with a larger residue therefore facilitating interaction with amphipathic drug molecules. E285K mutation will lead favorable interactions with nearby negatively charged residues such as D296, D292, and D301.

## 4 Discussion

We analyzed a total of 248 mutations present in ABCG2, ABCB1, and ABCC1 transporters. The position and physicochemical characteristics of the mutations were investigated to understand their possible impact on the function of transporters and its surroundings. Mutations present inside the binding pocket or channel are likely to affect the binding of substrate molecules. A drug molecule that can competitively bind in the pocket of these transporters with high affinity could block the passage for its natural substrates. This in turn will suppress the efflux of drug molecules from the cell, which is usually how cancer cells showcase multidrug resistance. It was observed that the frequency of mutations is also high in the membrane-facing regions. We believe this is because these mutations are involved in modulating the flexibility of the transmembrane and, in turn, affecting its surroundings, which in turn can affect the lipid and lipid raft composition of the cell membrane in the process. Change in the membrane composition affects the fluidity and surface curvature, which in turn is important for drug transport and efflux.

Existing inhibitory drugs such as tariquidar known to bind and inhibit P-glycoprotein (ABCB1) were studied for its inhibitory activity and also ABCB1 MDR activity (Ahmed et al., 2022). Discovery of inhibitor drugs for these transporters to tackle MDR has been a challenge. The phenomenon of MDR has been a major challenge for pharmaceutical industry. ABC transporters are often involved in drug efflux in diverse cell types; posing significant challenge in targeting various cancers. The binding sites of ABCB1, ABCC1 and ABCG2 are highly flexible and known to bind and transport diverse compounds including anti-cancer drugs. While this property helps these transporters to perform various important physiological activities, its overexpression has become one of the major cause for MDR in different cancer types. Targeting binding sites in these transporters is generally considered an attractive strategy to overcome drug resistance. However, the mutational analysis presented in this study, also suggests the importance of alternative strategies for addressing the chemoresistance by targeting the transmembrane and ATP binding domains for drug discovery.

Targeting of these specific residues to design an appropriate drug that can target both the binding region in the cavity and the transmembrane region have the potential to overcome MDRs. Designed inhibitors hypothetically will block the pathway, which will restrict the passage for the drug to be thrown out and will also prevent conformational and biochemical changes due to transmembrane mutations.

Research has shown that certain mutations can help reverse the direction of drug transport in these ABC transporters, which also have great potential in preventing MDR in cancer cells (Sajid et al., 2020). A group of 14 conserved residues (seven in both TMHs 6 and 12) were replaced with alanine and generated a mutant that lost the ability to pump most of the substrates tested out of cancer cells, but this reversed the direction of the pump, and the substrate was rather imported. It was able to import four substrates, including Rhodamine-123 and the taxol derivative flutax-1 (Kerb et al., 2001; Uitto, 2005; Seeger and van Veen, 2009; Kueppers et al., 2013; Kadioglu et al., 2020; Sajid et al., 2020).

## 5 Conclusions and future aspects

We performed a comprehensive analysis of mutations present in ABCB1, ABCC1, and ABCG2 transporters for endometrial cancer. The key mutations have been identified in these transporters and their possible functional impact on the drug binding, transport, and efflux has been discussed at the molecular level. The molecular level explanation of the impact of key mutations identified in ABCB1, ABCC1, and ABCG2 would be important in understanding the mechanisms of chemoresistance, design, and discovery of ABC inhibitors and therapeutic molecules. The key mutations identified in ABCB1, ABCC1, and ABCG2 can also be explored to reengineer these transporters from drug efflux transporters to drug importers to tackle chemoresistance.

Comparison of these mutations with ABC transporters expressed in normal endometrial tissue could be a potential area of exploration, which is currently unavailable on open access platforms. Additionally, there are no curated datasets for mutations present in all three ABC transporters to compare with cancerous and relapse-tissue samples from same patient pre- and post-chemotherapy. Due to the absence of mutational data from normal endometrial tissue (non-cancerous) for the ABC transporters, it is currently out of scope of this work to compare the mutational status between cancerous and normal endometrial tissues. Detailed analysis on these key aspects is important for understanding the intrinsic and acquired resistance and subsequent designing of inhibitors and therapeutic molecules.

## Data Availability

The original contributions presented in the study are included in the article/Supplementary Material; further inquiries can be directed to the corresponding authors.
